# Influence of mydriasis on optical coherence tomography angiography imaging in patients with age-related macular degeneration

**DOI:** 10.1371/journal.pone.0223452

**Published:** 2019-10-04

**Authors:** Viktoria C. Brücher, Jens J. Storp, Laura Kerschke, Pieter Nelis, Nicole Eter, Maged Alnawaiseh

**Affiliations:** 1 Department of Ophthalmology, University of Muenster Medical Centre, Muenster, North Rhine-Westphalia, Germany; 2 Department of Biometry and Clinical Research, University of Muenster Medical Centre, Muenster, North Rhine-Westphalia, Germany; Faculty of Medicine of the University of Porto, PORTUGAL

## Abstract

**Purpose:**

To evaluate the effect of topical mydriatic eye drops on optical coherence tomography angiography (OCTA) parameters in patients with age-related macular degeneration (AMD).

**Methods:**

27 eyes of 27 patients suffering from AMD were included in this cross-sectional study. Patients with ≥-4.5 diopters spherical equivalent, corneal opacities or dense cataract preventing high-quality imaging were excluded. Whole-en-face scans of the superficial capillary plexus (SCP) and deep capillary plexus (DCP) in the central 3x3mm foveal region as well as whole-en-face and peripapillary scans of the radial peripapillary capillaries (RPC) were generated using OCTA (AngioVue®, Optovue). Imaging was first conducted with patients’ eyes in miosis, then in mydriasis after instillation of a dilating eye drop (*0*.*5% tropicamide*, *2*.*5% phenylephrine-HCl*). Main outcome measures were flow density (FD), foveal avascular zone (FAZ), signal strength index (SSI) and motion artifact score (MAS).

**Results:**

Our results reveal that in AMD patients there is no significant difference between FD measurements taken in miosis and those taken in mydriasis around the SCP (p = 0.198), DCP (p = 0.458), RPC whole-en-face (p = 0.275) and RPC peripapillary (p = 0.503). Measurements taken in these two states appear to be equivalent for assessment of FD (90%CI within ± 0.05). No significant difference was found either in the area of the FAZ (p = 0.338) or in the SSI (p = 0.371) before and after the instillation of tropicamide/phenylephrine. MAS was significantly lower after the application of mydriatic eye drops (p = 0.003).

**Conclusions:**

Our findings reveal that neither measurements of FD nor measurements of the FAZ area changed significantly in AMD patients after the application of tropicamide/phenylephrine. Since MAS improved significantly in dilation, mydriatic examination is recommended. Nevertheless, a comparison of OCTA metrics from images taken with different pupil states (miosis versus mydriasis) is valid for clinical trials.

## Introduction

A new imaging technology, optical coherence tomography (OCT) angiography (OCTA) has recently been gaining attention in scientific research [1,2]. It has been evaluated in different ocular and systemic diseases and is gradually being incorporated into routine ophthalmologic practice. OCTA produces structural images of the blood flow within the retinal and choriocapillaris plexuses by combining the properties of the well-established OCT with a motion contrast feature [2]. It can visualize retinal and choroidal vessels in different layers of the retina and choroid as well as pathological neovascularization [3,4]. Moreover, OCTA allows quantitative evaluation of retinal and optic nerve head blood flow. The repeatability and reproducibility of quantitative OCTA metrics have been evaluated in depth in various recent studies in patients with healthy eyes and in patients with different ocular diseases [5–11].

Many studies have investigated factors influencing OCTA metrics such as motion artifacts and image quality [12–16], age and gender [17–22], spherical equivalent and axial length [13–25] and systemic diseases [26–31] and thereby indicated how to apply and interpret OCTA most adequately. There is very little literature on the influence of mydriasis on quantitative OCTA parameters such as flow density (FD), foveal avascular zone (FAZ), signal strength index (SSI) or motion artifact score (MAS) for image quality.

Cheng et al. [32] have looked into the effect of topical mydriatic eye drops on OCTA metrics in a young healthy control group (mean age: 30.13 ± 4.12 years). However, as patients in clinical practice are usually older and have visual impairment, quantitative OCTA metrics might be affected differently. The aim of this study was therefore to evaluate the impact of the state of the pupil on quantitative OCTA metrics in age-related macular degeneration (AMD) patients. We achieved this aim and present our findings here.

## Methods

### Patients

The data in this study were derived from 27 eyes of 27 patients suffering from early, intermediate and advanced AMD (AREDS Classification) [33,34]. Patients were examined at the Department of Ophthalmology, University of Muenster Medical Center between 3^rd^ November 2017 and 15^th^ December 2017. All procedures performed in this study were in accordance with the ethical standards of the institutional and national research committee (approved by the ethics committee of the University of Muenster, Germany) and with the 1964 Helsinki declaration and its later amendments. Written informed consent was obtained from all individual participants included in the study. Patients with ≥-4.5 diopters spherical equivalent, corneal opacities or dense cataract preventing high-quality imaging or Parkinson´s disease were excluded. OCTA images with an SSI of < 42 were also excluded. All patients underwent a standard ophthalmic examination including refraction, best corrected visual acuity (BCVA), measurement of intraocular pressure (Goldmann applanation tonometer), anterior segment examination, and fundus examination.

### Examination

**Fig 1:**
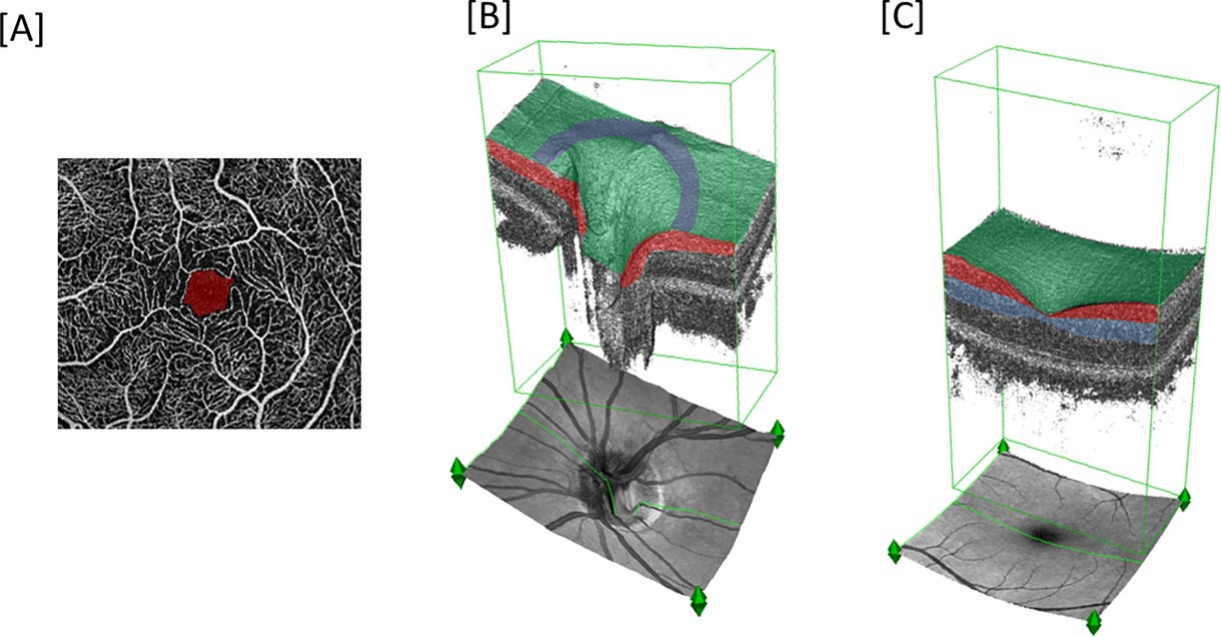
(A) Typical optical coherence tomography angiography (OCTA) image of healthy macular region with superimposed foveal avascular area (FAZ) (red). (B) healthy optic nerve head (ONH) 3D reconstruction image with superimposed color coding representing segmentation layers and topical regions: radial peripapillary capillary (RPC) layer (red), whole-en-face region (green), peripapillary region (purple). (C) macular 3D-reconstruction image with superimposed color coding representing segmentation layers and topical regions: superficial capillary plexus (SCP) (red), deep capillary plexus (DCP) (blue), whole-en face region (green).

Imaging was conducted with the RTVue XR Avanti system (Angiovue/RTVue-XR Avanti optical coherence tomograph, Optovue Inc., Fremont, USA), which applies the split-spectrum amplitude-decorrelation angiography (SSADA) algorithm to extract flow data. The macula was imaged in a 3x3 mm square, whereas the optic disc was imaged in a 4.5x4.5 mm square. The superficial capillary plexus (SCP) was generated from the inner limiting membrane with an offset of 0 μm to the inner plexiform layer with an offset of -9 μm. The deep capillary plexus (DCP) was generated from the inner plexiform layer with an offset of -9 μm to the outer plexiform layer with an offset of 9 μm (Fig 1). The radial peripapillary capillaries (RPC) were generated from the internal limiting membrane with an offset of 0 μm to the nerve fiber layer posterior boundary within the 4.5x4.5mm optic nerve head (ONH) acquisition. Peripapillary FD was measured within the RPC using a 0.75mm wide annulus extending from the optic disc boundary (Fig 1). The flow density data of the SCP and DCP (whole-en-face) and of the RPC layer of the ONH (whole-en-face and peripapillary) were analyzed. The area of the FAZ in mm^2^ was calculated in the superficial capillary plexus and was also evaluated automatically by integrated software Angioanalytics (Fig 1).

OCTA imaging was done in two imaging sessions for each patient under the same conditions in the same location by an expert examiner (once before and once after pupil dilation), each composed of three acquisitions. The same expert examiner administered the pupil-dilating drug (*0*.*5% tropicamide*, *2*.*5% phenylephrine-HCl*). The second imaging session was performed 15 minutes after the administration of one drop of tropicamide/phenylephrine. Additionally, the onset of dilation was checked visually by the expert examiner before continuing with the second imaging session. Furthermore, subjects had to rest for at least 10 minutes before imaging was performed, as recommended by Alnawaiseh et al. [35]. We selected the image with the fewest motion artifacts, correct segmentation and highest SSI.

The motion artifact score, as described by Lauermann et al. [12] for macular OCTA images, was evaluated before and after the application of mydriatic eye drops by two independent examiners; the mean was taken for analysis. The two independent graders were unmasked for the images. The MAS score identifies five major imaging artifacts (quilting, displacement, vessel doubling, stretch artifacts, blink lines) and the presence/absence or extent of each artifact defined the attribution to OCTA MAS 1 through 4 [12].

### Statistical analysis

Statistical analyses were performed using IBM^®^ SPSS^®^ Statistics (Version 25, 2017).

The primary endpoint of this study was the FD at the SCP and DCP in the central 3x3mm foveal region and the whole-en-face RPC and peripapillary RPC area. The primary aim of the project was to establish whether or not there is a change in FD in the SCP and DCP and in the whole-en-face RPC and peripapillary RPC area with different pupil states (miosis versus mydriasis). In order to control a multiple significance level of 5%, a Bonferroni correction was applied, i.e. each test was carried out at the local level of 5%/4 = 1.25%. All further analyses were exploratory, not confirmatory, and were interpreted accordingly. P-values ≤ 0.05 were considered statistically significant.

Patient characteristics were described by standard descriptive statistical measures. Continuous variables are presented as mean ± standard deviation (SD). Categorical variables are reported as absolute frequencies. The distribution of the data was checked with the Kolmogorov-Smirnov test. FD and SSI showed normal distribution and were then compared using the paired t-test. FAZ and MAS showed non-normal distribution and were therefore analyzed using the paired Wilcoxon-test.

Parameters for which no differences could be detected before and after administration of tropicamide/phenylephrine were investigated further based on the interval inclusion test for equivalence which is equivalent to Schuirmann’s [36] two one-sided tests approach (TOST). The test was performed on the relative differences between outcome measures using a pre-determined tolerance range of -0.05 to 0.05. To derive the test decision, the two-sided 90% confidence interval for the difference of means was calculated for each outcome measure. Where the respective confidence interval lay within the above-mentioned tolerance range, the two measurement conditions (miosis and mydriasis) were considered equivalent, meaning that the results generated under the two conditions did not differ in any clinically relevant way.

## Results

Characteristics of the study population are summarized in Table 1.

**Table 1:** Characteristics of study population.

	study subjects
Age (y, mean ± SD)	78.07 ± 12.31
Gender (M/F)	10/17
Visual acuity (LogMAR, mean ± SD)	0.54 ± 0.46
Spherical equivalent (D, mean ± SD)	0.24 ± 1.42
IOP (mmHg, mean ± SD)	15.96 ± 3.42
Early AMD	1 (3.70%)
Intermediate AMD	7 (25.93%)
Advanced AMD	19 (70.37%)
• CNV	15 (78.95%)
• cRORA	4 (21.05%)
Systemic medication	
• Sympathomimetic/parasympatholytic drug	0
• Sympatholytic/parasympathomimetic drug	0
• Antidiabetic therapy	4
• Antihypertensive drug	13
• Tamsulosin	1
Systemic diseases	
• Stroke	2
• Diabetes mellitus	4

SD (standard deviation), y (years), M (male), F (female), LogMar (logarithm of the minimum angle of resolution), D (diopter), mmHg (millimeters of mercury), IOP (intraocular pressure), age-related macular degeneration (AMD), choroidal neovascularization (CNV), complete retinal pigment epithelium and outer retinal atrophy (cRORA).

Neither SCP (p = 0.198) nor DCP (p = 0.458) and neither RPC whole-en-face (p = 0.275) nor RPC-peripapillary (p = 0.503) appeared to be significantly different in the states of miosis and tropicamide/phenylephrine-induced mydriasis (Table 2).

**Table 2:** Pre- and post-dilation FD results.

	pre-dilation (±SD)	post-dilation (±SD)	p-value
**3x3mm foveal acquisition**			
SCP, whole-en-face	45.19 ± 4.37	44.56 ± 3.45	0.198
DCP, whole-en-face	51.36 ± 3.90	51.00 ± 4.10	0.458
**4.5x4.5mm ONH acquisition**			
RPC, whole-en-face	49.16 ± 4.70	49.66 ± 4.95	0.275
RPC, peripapillary	56.33 ± 5.62	56.33 ± 5.90	0.503

Results for pre- and post-dilation flow density (FD) of superficial capillary plexus (SCP) and deep capillary plexus (DCP)(3x3mm foveal acquisition) and radial peripapillary capillaries (RPC) layers (4.5x4.5mm ONH acquisition). In accordance with Bonferroni, correction outcomes are only considered significant where p<0.0125. Acronyms: before dilation (pre-dilation); after dilation (post-dilation); standard deviation (SD), millimeter (mm), optic nerve head (ONH).

There was no significant difference, neither in the FAZ (before: 0.29 ± 0.13 mm^2^; after: 0.31 ± 0.13 mm^2^, p = 0.338) nor in the SSI (before: 58.40 ± 8.82; after: 59.20 ± 7.10; p = 0.371), before and after instillation of tropicamide/phenylephrine.

**Fig 2:**
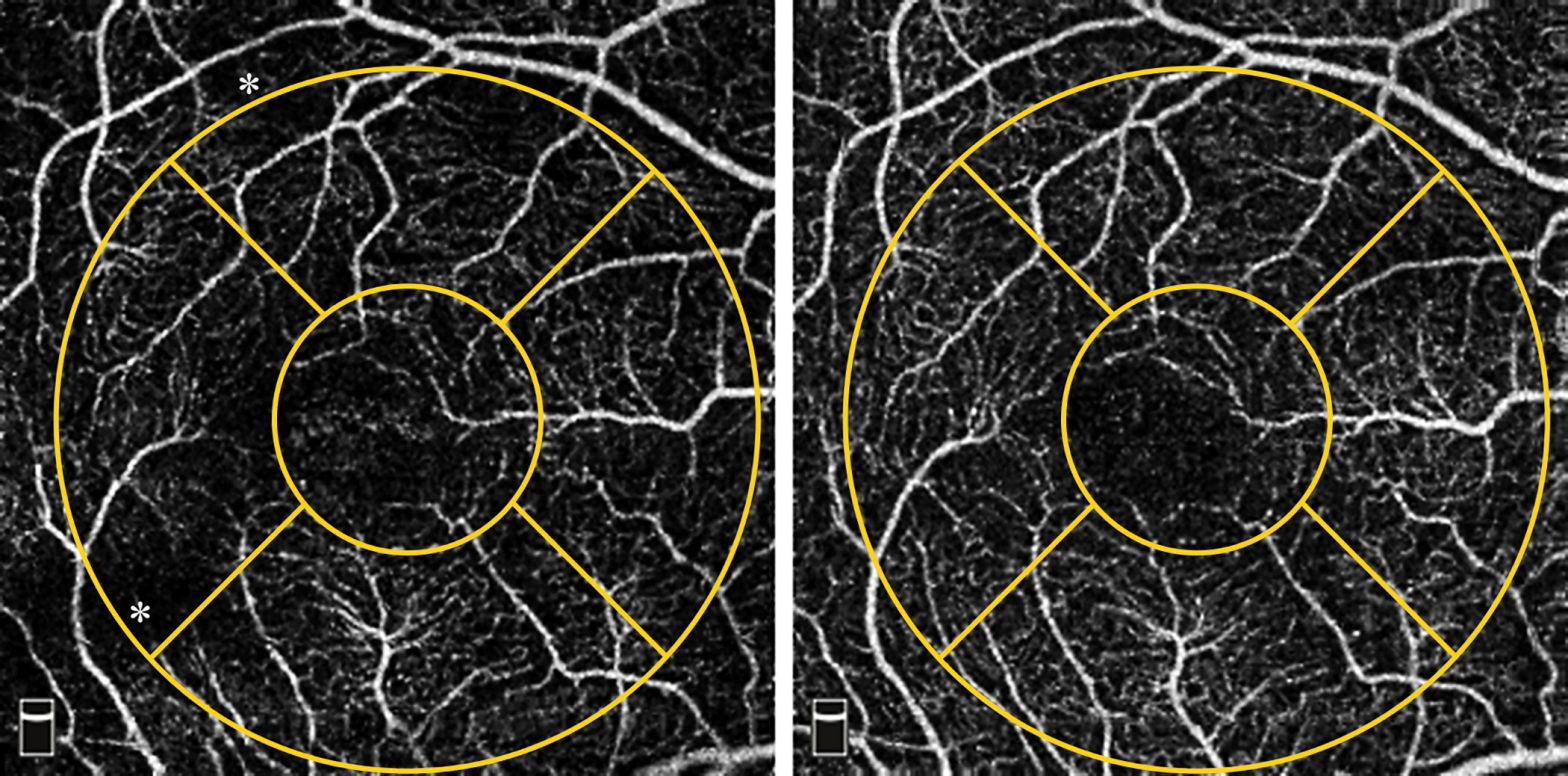
Example of reduction of artifacts evaluated by motion artifact score (MAS) before (A1) and after (A2) pupil dilation. A1 shows a MAS of 2 with slight-moderate quilting (examples marked with asterisks), A2 shows a MAS of 1: no quilting, absence of all other artifacts. Asterisks: focal changes in flow density (FD) in regions where artifacts are present.

The motion artifact score was significantly lower after dilation (before: 2.24 ± 0.93; after: 1.80 ± 0.85; p = 0.003) (Fig 2). Complete agreement between the two examiners was found in 16 cases (59.26%) pre-dilation and in 15 cases (55.56%) post-dilation.

Statistical results for the equivalence tests are displayed in Table 3. We defined a deviation of no more than ± 5% between parameter outcomes in the two states of the pupils as equivalent. The results for the four regions tested show that FD measurements in miosis and mydriasis provide outcomes within the pre-defined equivalence interval (-0.05–0.05) (Table 3).

**Table 3:** Results for the 90%-confidence intervals.

	90%-CI
**3x3mm foveal acquisition**	
SCP, whole-en-face	**-0.011–0.033**
DCP, whole-en-face	**-0.013–0.026**
**4.5x4.5mm ONH acquisition**	
RPC, whole-en-face	**-0.029–0.009**
RPC, peripapillary	**-0.024–0.013**

Results for the 90%-confidence intervals of the relative differences between measurements in miosis and mydriasis in their respective locations for age-related macular degeneration (AMD) patients. Statistically significant results (i.e. inclusion of the respective CI in the pre-defined tolerance range of ± 5%) indicate equivalence of the two measurement conditions and are marked in bold. Acronyms: confidence interval (CI), superficial capillary plexus (SCP), deep capillary plexus (DCP),radial peripapillary capillaries (RPC), millimeter (mm), optic verve head (ONH).

When analyzed using the interval inclusion test for equivalence, both FAZ and SSI exceeded the preset tolerance range of ± 0.05 in the negative half (FAZ: -0.288–0.028; SSI: -0.056–0.012), indicating that FAZ and SSI results were on average higher in mydriasis than in miosis. Therefore, while not being significantly different (p = 0.338; p = 0.371), no equivalence of FAZ and SSI measurements in the two states of the pupils could be shown, either.

## Discussion

Over the years OCTA has become the focus of an increasing number of studies, several of which do not mention whether the state of the pupil was considered in their trials, while comparing quantitative OCTA values taken in possibly different pupil states. This has raised the question, whether quantitative OCTA results are different in miosis and mydriasis and whether they are comparable to each other in various pathologies, such as AMD. Thus, we analyzed OCTA imaging before and after application of a mydriatic drug (*0*.*5% tropicamide*, *2*.*5% phenylephrine-HCl*) and evaluated FD, FAZ, SSI and MAS.

Our findings point to an equivalence of FD evaluation in miosis and mydriasis with tropicamide/phenylephrine in AMD patients. The mean MAS improved significantly after the tropicamide/phenylephrine mixture had been instilled.

Several clinical trials have investigated the influence of mydriasis on OCT [37–46]. Some authors report a significant difference in OCT-based parameters between pre- and post-dilation [39–41,46], whereas most studies describe no significant difference between pre- and post-dilation measurement results [37,38,42–45].

A study by Cheng et al. [32] analyzed OCTA images of 16 eyes of 8 healthy patients undergoing pupil dilation using different eye drop mixtures. First pupil dilation was performed using tropicamide eye drops. The following week, study subjects received a phenylephrine/tropicamide mixture. The authors applied three eye drops at 10-minute intervals and imaged 10 minutes after the last eye drop. When tropicamide eye drops were used, there were no significant differences in peripapillary, perifoveal or parafoveal measurements or in FAZ outcomes. Analysis of results with the phenylephrine/tropicamide mixture revealed no significant differences for the perifoveal and parafoveal layers or for the FAZ. However, peripapillary layers did show a slight but significant reduction in FD after dilation (p = 0.034). The authors concluded that topical phenylephrine reduced the retinal vessel density at peripapillary locations [32].

We, however, did not observe any significant difference in FD or FAZ pre- and post-dilation using a phenylephrine/tropicamide mixture.

One possible explanation for these contradictory findings could be the number of eye drops applied. In the study presented by Cheng et al. (n = 8) the mydriatic eye drops were applied every 10 minutes, for a total of three instillations. We chose to apply mydriatic eye drops only once, then to wait 15 minutes and check for proper dilation, as is done in clinical practice. It is reasonable to assume that multiple instillations of phenylephrine/tropicamide eye drops could affect FD more significantly than just one single instillation.

Our studies are corroborated by Polak et al. [47], who found no relationship between phenylephrine and retinal vessels. Retinal vessel diameter was measured using a Zeiss retinal vessel analyzer (Zeiss FF 450, Jena, Germany). Retinal leucocyte velocity, flow and density were measured with the blue field entoptic technique. The authors found that neither placebo nor phenylephrine influenced retinal hemodynamics.

Among the factors influencing FD and FAZ, artifacts as well as image quality have proved to be of critical importance. Several investigations have shown that these artifacts may affect the accuracy of measurements [12–16]. To our knowledge, there is no study-based consensus or recommendation stating which state of the pupil provides the fewest motion artifacts and highest image quality and is therefore most favorable for OCTA imaging.

Previous studies have reported various cutoff values for the signal strength index [48–50]. Al-Sheik and co-workers elaborated on the effect of different levels of SSI on a quantitative analysis of OCTA images in healthy eyes. The study group found the frequency of artifacts to be higher and the repeatability of FD measurements to be lower in images of reduced quality [51]. In our study, no significant difference was found between SSI results pre- and post-dilation.

As Lauermann et al. pointed out, “a high SSI level and a high repeatability of FD measurements do not necessarily preclude the presence of motion artifacts, even if active eye-tracking is used” [52]. Thus, there is pressing need for other quantitative parameters. The motion artifact score described by Lauermann et al. provides information on OCTA image quality with regard to image artifacts. Our results reveal that pre-dilation MAS was significantly higher than post-dilation MAS, translating to fewer and/or less severe motion artifacts in mydriasis. One explanation for this might be that the tracker of the OCTA device fails due to a worse fundus image in miosis. Further studies using different OCTA devices are needed to investigate this issue.

Even though the overall FD does not change in this study, it is possible that focal changes are present due to artifacts (Fig 2). This is of special relevance in the many studies where angiograms are examined topographically in AMD [53–55] or for the detection of ischemic areas in diabetic retinopathy [56,57].

Our study has some limitations. Firstly, we analyzed only a small cohort (n = 27), which nevertheless represents the largest cohort in the literature to date. Nevertheless the results cannot be extrapolated to patients with media opacities. Secondly, we analyzed only AMD patients, which prohibits us from applying conclusions drawn from this trial to other patient groups. Thirdly, we merely analyzed the effect of a tropicamide/phenylephrine mixture on OCTA imaging. We cannot therefore make statements about the effects of mydriatic drugs on OCTA imaging in general. Other drugs, such as atropine might behave differently. Further studies investigating the effects of a variety of drugs in patients with different eye diseases would be helpful. Furthermore, as in clinical practice, this study distinguished between the states of miosis and mydriasis only by visual assessment. It would be interesting in further studies to measure exact pupil size and analyze the impact of this factor on OCTA. Lastly, some study participants took systemic medication, which might have affected ocular circulation and thereby FD evaluation.

In conclusion, this study suggests that there is no significant difference between OCTA-driven results for FD, FAZ or SSI after instillation of a tropicamide/phenylephrine mixture in AMD patients. FD measurements can even be considered equivalent in miosis and mydriasis. These findings could be of significance in clinical practice and scientific research. We would recommend generating OCTA images in mydriasis rather than in miosis, as MAS was significantly lower in mydriasis than in miosis.
